# Quorum-sensing microbial dark matter enables core functions in wastewater microbiomes

**DOI:** 10.1093/ismeco/ycag264

**Published:** 2026-09-16

**Authors:** Qiuju Liu, Han Su, Sai Gong, Ying Jin, Yingzheng Fan, Mengxiong Wu, Jianhua Guo, Xinkun Ren, Jinfeng Wang

**Affiliations:** State Key Laboratory of Water Pollution Control and Green Resource Recycling, School of the Environment, Nanjing University, Nanjing 210023, Jiangsu, China; State Key Laboratory of Water Pollution Control and Green Resource Recycling, School of the Environment, Nanjing University, Nanjing 210023, Jiangsu, China; Institute for the Environment and Health, Nanjing University, Suzhou Campus, Suzhou 215163, Jiangsu, China; State Key Laboratory of Water Pollution Control and Green Resource Recycling, School of the Environment, Nanjing University, Nanjing 210023, Jiangsu, China; State Key Laboratory of Water Pollution Control and Green Resource Recycling, School of the Environment, Nanjing University, Nanjing 210023, Jiangsu, China; State Key Laboratory of Water Pollution Control and Green Resource Recycling, School of the Environment, Nanjing University, Nanjing 210023, Jiangsu, China; State Key Laboratory of Water Pollution Control and Green Resource Recycling, School of the Environment, Nanjing University, Nanjing 210023, Jiangsu, China; Australian Centre for Water and Environmental Biotechnology (ACWEB, Formerly AWMC), The University of Queensland, Brisbane, St. Lucia, Queensland, 4072, Australia; College of Engineering and Applied Sciences, Nanjing University, Nanjing 210023, Jiangsu, China; State Key Laboratory of Water Pollution Control and Green Resource Recycling, School of the Environment, Nanjing University, Nanjing 210023, Jiangsu, China

**Keywords:** metagenome, metagenome-assembled genomes, genome-scale metabolic modeling, activated sludge, co-occurrence network, machine learning, cross-feeding, complementarity network, signaling molecule, keystone

## Abstract

Quorum-sensing bacteria (QSB) play a crucial role in coordinating microbial activities in activated sludge. However, our knowledge is primarily based on cultivated or taxonomically classified species, neglecting microbial dark matter (MDM), which comprises lineages lacking reference genomes and is prevalent in wastewater communities. Herein, 3569 high- and medium-quality metagenome-assembled genomes were obtained from 69 full-scale wastewater treatment plants. A total of 1289 MDM lineages were identified to possess quorum sensing (QS) capabilities, designated as MDM-QSB. Among them, 127 novel genera were identified. These organisms possess larger genomes enriched in genes for energy metabolism and environmental information processing, allowing a broader ecological niche compared to non-QS MDM (*P* < .001). They occupy central positions in co-occurrence networks and are predicted as dominant keystones by a deep learning model. Their robust metabolic potential and network keystone position enable coordinated community-level metabolic responses, as evidenced by stronger carbon, nitrogen, phosphorus, and sulfur element cycling functions and higher associations with chemical oxygen demand, total nitrogen, and ammonium nitrogen removal (*P* < .05). Furthermore, genome-scale metabolic modeling reveals they engage in extensive cross-feeding with nitrifiers, denitrifiers, and polyphosphate-accumulating organisms, supplying metabolites such as protoheme and serine alongside QS signals. Together, these findings identify MDM-QSB as previously overlooked multifunctional hubs of activated sludge microbiomes. Given their crucial roles in community stability and process performance, incorporating these previously overlooked populations into monitoring and control frameworks is expected to improve the resilience and predictability of biological wastewater treatment processes.

## Graphical abstract

**Graphical abstract:**
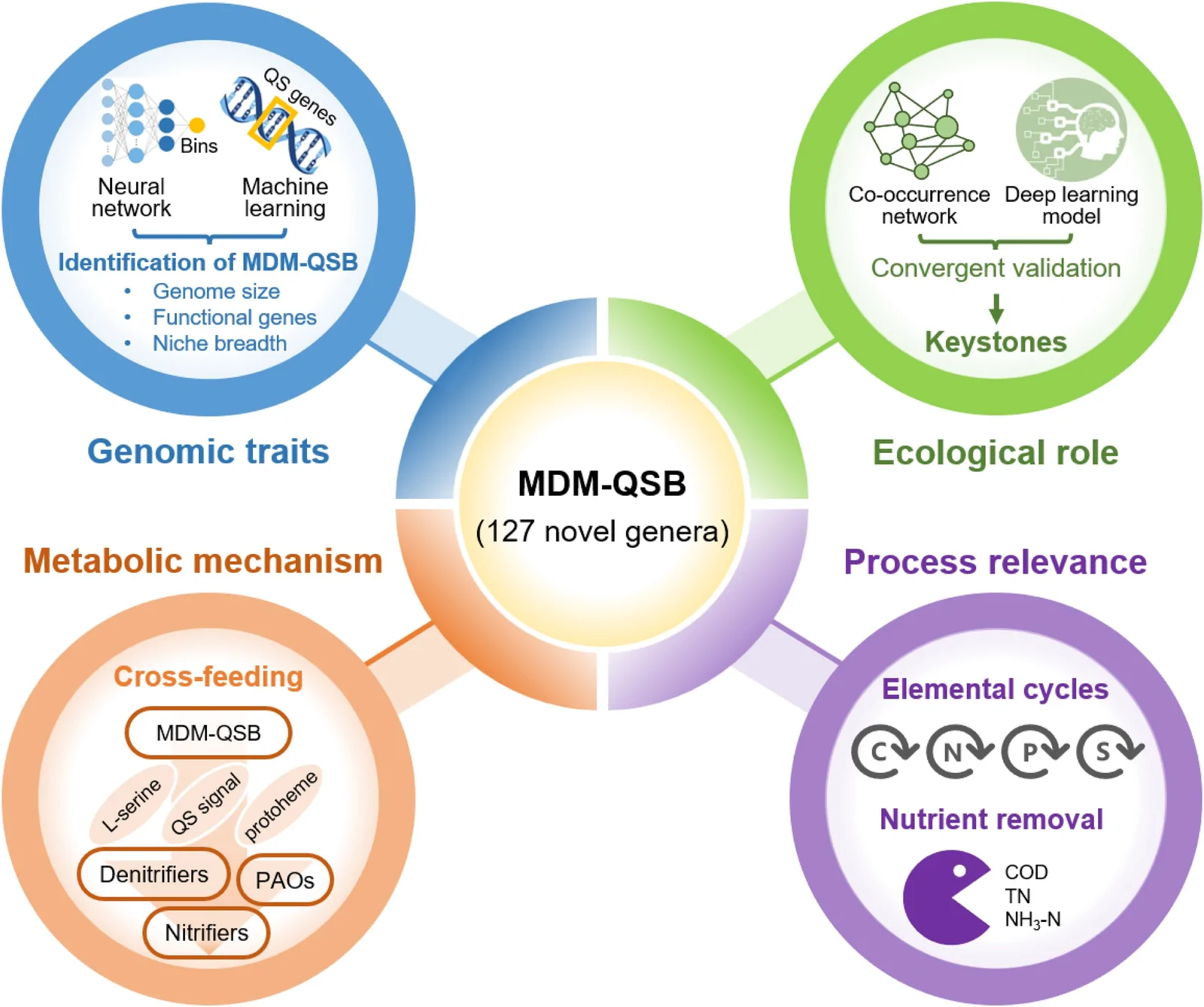

## Introduction

Quorum-sensing bacteria (QSB) are recognized as key mediators of microbial coordination in activated sludge systems, where they contribute to community stability through cell–cell communication, stress response modulation, and collective regulation of metabolic activities [1]. Current models of quorum sensing (QS) in engineered microbiomes are based almost exclusively on genomically characterized or culturable taxa. This framework implicitly assumes that chemical signaling is orchestrated by a limited set of known organisms, thereby overlooking the potential contributions of microbial dark matter (MDM), which constitutes a large fraction of the community and comprises organisms without reference genomes that remain unclassified in standard databases [2]. Although metagenomic sequencing has revealed extensive sequence diversity in wastewater treatment systems, the functional implications of MDM in intercellular communication remain unexplored. In particular, it is unknown whether MDM lineages encode QS systems, whether their signaling molecules can be detected by functionally important guilds, and whether such interactions influence nitrogen and phosphorus removal processes in activated sludge.

Recent genome-resolved studies have begun to uncover the metabolic potential of MDM in engineered ecosystems. These efforts have identified novel lineages that expand the known phylogenetic breadth of bacterial diversity [3–5] and revealed MDM genomes enriched in pathways for degrading complex organic pollutants [6] or performing redox transformations typically requiring syntrophic partnerships [7]. In activated sludge, MDM can account for up to 50% of the microbial community [8], and its genomic content suggests a disproportionate contribution to functional novelty relative to their abundance [9–12]. Nevertheless, existing analyses have focused primarily on autonomous metabolic traits, with little attention to the capacity of MDM to engage in intercellular communication. The possibility that MDM participate in QS and thereby influence the physiology of partner taxa remains unexamined. No evidence currently exists that MDM-derived signals are perceived by core functional guilds such as nitrifiers, denitrifiers, or polyphosphate-accumulating organisms (PAOs), nor that such signaling modulates gene expression or metabolic fluxes in partner taxa. Bridging this gap is critical to understanding whether phylogenetically obscure microbes serve not only as metabolic actors but also as coordinators of community behavior in complex microbiomes.

Resolving whether MDM participate in QS requires high-quality genomic reconstructions that reliably capture their signaling machinery [5, 13]. However, many MDM taxa occur at low relative abundance in activated sludge, rendering their genomes difficult to recover with standard metagenomic binning approaches [14–16]. Conventional pipelines often yield fragmented or chimeric metagenome-assembled genomes (MAGs) for rare populations, which compromises the detection of complete QS loci such as synthase-receptor gene pairs and autoinducer transport systems that are essential for functional inference [17, 18]. Consequently, the underrepresentation of MDM in current genome catalogs may have led to a systematic underestimation of their role in chemical communication.

To address this limitation, we employed an integrated metagenomic binning framework that enables high-confidence recovery of MAGs from low-abundance lineages. Using this approach, we recovered 3569 high-quality MAGs from activated sludge metagenomes and identified a distinct group of QSB within MDM, designated MDM-QSB, through machine learning-based prediction. We then characterized their genomic potential, topological roles in co-occurrence networks, and metabolic interdependencies with core functional guilds through functional enrichment and genome-scale metabolic modeling (GSMM). This work demonstrates that MDM-QSB are not functionally inert bystanders but ecologically central populations that contribute to community functions through coordinated signaling and metabolic support of key nitrogen- and phosphorus-cycling taxa, thereby expanding our understanding of the role of MDM in engineered microbiomes. Incorporating MDM-QSB into monitoring and control strategies could improve the predictability and resilience of wastewater treatment processes.

## Materials and methods

### Sampling

Four hundred fourteen activated sludge samples were collected from 69 wastewater treatment plants (WWTPs) across China. One to two samples were randomly selected from each WWTP, and thus 96 sludge samples were obtained for metagenome sequencing. Of these, 43 sludge samples were from the anaerobic/anoxic/oxic process, 15 each from the anoxic/oxic, integrated fixed-film activated sludge, and oxidation ditch process, and 8 from the cyclic activated sludge system. This sampling design was intended to cover the major full-scale activated sludge process configurations rather than a single process type, thereby ensuring a broad and representative sampling of wastewater treatment systems. Specifically, 40 ml of sludge was collected and transferred into Corning centrifuge tubes and stored in a −80°C refrigerator following transportation to the laboratory via dry ice boxes. In addition, 20 l of wastewater was collected, and the water quality, including chemical oxygen demand (COD), total nitrogen (TN), ammonium nitrogen (NH_3_-N), and total phosphorus (TP), was measured immediately after transportation to the laboratory via a low-temperature compartment.

### DNA extraction and metagenomic sequencing

Total DNA was extracted from the activated sludge samples using the Mag-Bind® Soil DNA Kit (Omega Bio-tek Inc., GA, USA) following the manufacturer’s instructions. The concentration and purity of extracted DNA were determined with NanoDrop 2000 (Thermo Fisher Scientific, Wilmington, USA). The metagenomic sequencing was performed by Majorbio Bio-pharm Technology Co., Ltd. (Shanghai, China). DNA extract was fragmented to an average size of about 350 bp using Covaris M220 apparatus (Gene Company Limited, China). The construction of the paired-end library was then undertaken using NEXTFLEX® Rapid DNA-Seq (Bioo Scientific, Austin, TX, USA). Adapters containing the full complement of sequencing primer hybridization sites were ligated to the blunt end of fragments. Paired-end sequencing was performed on Illumina NovaSeq X-plus (Illumina, San Diego, CA, USA) using NovaSeq 6000 S4 Reagent Kit v1.5 (300 cycles). Sequence data associated with this project have been deposited in the NCBI Database (Accession number: PRJNA1271453).

### Sequence data processing

Raw paired-end reads were initially filtered using fastp (v.0.23.4) [18] to detect and trim sequence adapters. Clean reads were individually and co-assembled using SPAdes (v.3.15.5) [19] under “—meta” mode into contigs specifying k-mer sizes of 21, 33, 55, and 77. Metagenomic binning was applied to both single-sample assembly and co-assembly using BASALT (v.1.1.0) [20]. This tool employs multiple binners including MetaBAT2, MaxBin2, CONCOCT, SemiBin2, and MetaBinner to produce initial bins, then uses neural networks to identify core sequences to remove redundant bins and refine non-redundant bins. This optimized binning strategy improved the number and quality of recovered genomes [20]. The quality of the recovered MAGs was evaluated using CheckM (v.1.0.2) [21]. Only MAGs satisfying medium-quality criteria (≥50% completeness and <10% contamination) or high-quality criteria (≥90% completeness and <5% contamination) were retained, yielding 3569 MAGs. Taxonomic identity was inferred with genome taxonomy database toolkit (GTDB-Tk (v2.5.4) [22] against GTDB release 220; genomes lacking species-level assignment were operationally defined as MDM. It should be noted that this definition is database-dependent and reflects the current limits of taxonomic classification rather than an absolute biological category. In practice, this category spans a continuum, ranging from unnamed species that belong to well-characterized genera with close cultured relatives (e.g. *Nitrospira*) to deeply branching lineages lacking cultured representatives at any rank. Truly undetected lineages that fall below the detection limit of metagenomic assembly and binning are not captured by this definition and remain outside the scope of this study. A maximum-likelihood phylogeny was reconstructed with the GTDB-Tk infer workflow. Genome-wide abundance was quantified by mapping quality-trimmed reads to each MAG with Bowtie 2 (v2.5.4) [23] and CoverM, followed by reads per kilobase per million mapped reads (RPKM) normalization to both genome length and library size. MAGs accounting for <0.01% of the community are referred to as rare taxa, those ≥0.01% as abundant species [24].

### Identification of MDM-QSB

Protein-coding sequences (CDSs) of 3569 high- and medium-quality MAGs were predicted with Prodigal (v2.6.3) [25]. QS proteins were identified with the ensemble machine-learning pipeline of Jin *et al*. [26]. Candidate peptides were scored independently by random-forest and k-nearest-neighbor classifiers, and only those receiving positive predictions from both models were retained. These two classifiers operate on amino acid composition features intrinsic to protein sequences rather than on taxonomic identity, supporting their applicability to MDM lineages lacking reference genomes. Each validated QS protein was then assigned to a QS language type and functionally classified as either a synthase or a receptor on the basis of conserved-domain architecture and evolutionary genealogy of genes: non-supervised orthologous groups (eggNOG)-mapper annotation. MDMs harboring these experimentally supported QS modules are hereafter referred to as MDM-QSB.

### Function analysis of metagenomes

The predicted CDSs were annotated with eggNOG-mapper (v2.1.12) [27] with database (v5.0.2) [28] under the mode of DIAMOND [29] for functional annotation. Relative abundance of genes was defined as the number of gene sequence reads. The obtained functional profiles of Kyoto encyclopedia of genes and genomes (KEGG) orthology (KO) terms and the genetic potential of a specific pathway were analyzed following the key gene families involved in C, N, P, and S cycles.

### Construction of species co-occurrence network

The species co-occurrence network was constructed using Spearman correlation analysis based on the RPKM abundance of 986 species using the Molecular Ecological Network Analysis Pipeline (http://ieg4.rccc.ou.edu/MENA/) [30]. Only direct ecological associations between different species were retained. The correlation threshold (*r* ≥0.89) was automatically determined using random matrix theory. Network topological parameters were calculated via the pipeline. The within-module connectivity (*Z_i_*) and among-module connectivity (*P_i_*) of each node were calculated to define their roles in the network: peripherals (*Z_i_* < 2.5, *P_i_* < 0.62), connectors (*Z_i_* < 2.5, *P_i_* ≥ 0.62), module hubs (*Z_i_* ≥ 2.5, *P_i_* < 0.62), and network hubs (*Z_i_* ≥ 2.5, *P_i_* ≥ 0.62). The latter three were regarded as keystone taxa.

We further validated keystones obtained from network topology using a deep learning-based keystone identification (DKI) model [31], which uses compositional neural ordinary differential equations (cNODE2) [32] to quantify the impact of subsequent species removal by learning the mapping between microbial composition and relative abundance. Specifically, the input to the DKI model is the RPKM-normalized species relative abundance matrix (samples × species), from which the model learns the embedding representation of each species and quantifies its structural importance for community stability. The model follows the default hyperparameter configuration as described in Wang *et al*. [31], including an embedding dimension of 64 and 200 training epochs.

### Construction of metabolic complementarity network

GSMMs for the 3569 MAGs were reconstructed with CarveMe (v1.6.4) as implemented in the iNAP 2.0 platform [33, 34], without applying gap-filling, so that all model reactions were inferred directly from genome annotations rather than introduced to enable growth on a predefined medium. PhyloMint metabolic complementarity index (MI*_Complementarity_*) was used to represent the cooperation potential of pairwise GSMMs or MAGs [35]. The metabolic complementarity network was delineated by Z-score outlier detection, employing a modified Z-score threshold of 2.698 [34]. Within this network, within-module connectivity ≥2.5 or among-module connectivity ≥0.62 was used to identify keystone taxa. Meanwhile, pairwise exchangeable metabolites between MAGs were predicted and classified according to a reported scheme [34].

### Statistical analysis and visualization

We characterized microbial communities using three complementary diversity metrics: Shannon index, Chao1 index, and Faith’s phylogenetic diversity [36]. The microbial niche breadth was determined using the R package vegan. To examine the relationships between microbial communities and their functions, we integrated species abundance data with four key pollutant removal rates, including COD, TN, NH_3_-N, and TP. The nonlinear association between species and the pollutant removal rates was quantified using the maximal information coefficient (MIC) [37]. Higher MIC values indicate stronger dependencies. Meanwhile, the statistical significance was assessed using 1000 bootstrap resamplings to generate 95% confidence intervals. Associations were considered significant (*P* < .05) when the lower MIC was greater than zero (*P* < .05). Furthermore, the phylogenetic relationship of MAGs was visualized using the iTOL platform (v6.9.1) [38]. The species co-occurrence network was constructed and visualized in Gephi (v0.10) [39]. Partial violin plots were generated using the R packages “ggpubr” and “ggh4x” to display data distributions effectively while maintaining visual clarity.

## Results

### Genomic and regulatory expansion in quorum sensing-capable microbial dark matter

Of the 3569 high- and medium-quality MAGs recovered from activated sludge, 1923 (53.9%) could not be assigned to any species-level taxon in GTDB release 220, indicating that a substantial fraction of the microbial community remains uncharacterized. Among these uncultivated taxa, 1289 were identified as possessing QS capability, defining them as MDM-QSB (Fig. 1a). This group constitutes 67.0% of the unclassified MAGs (Fig. 1b), highlighting the prevalence of QS functionality within MDM. Functional annotation revealed that 97 MDM-QSB MAGs (7.5%) encode only putative signal synthases, 894 (69.4%) contain only receptor proteins, and 298 (23.1%) possess both. The overwhelming dominance of receptor-only lineages indicates that most MDM-QSB do not produce signaling molecules but instead engage in eavesdropping (i.e. perception of exogenous autoinducers without synthesis).

**Figure 1:**
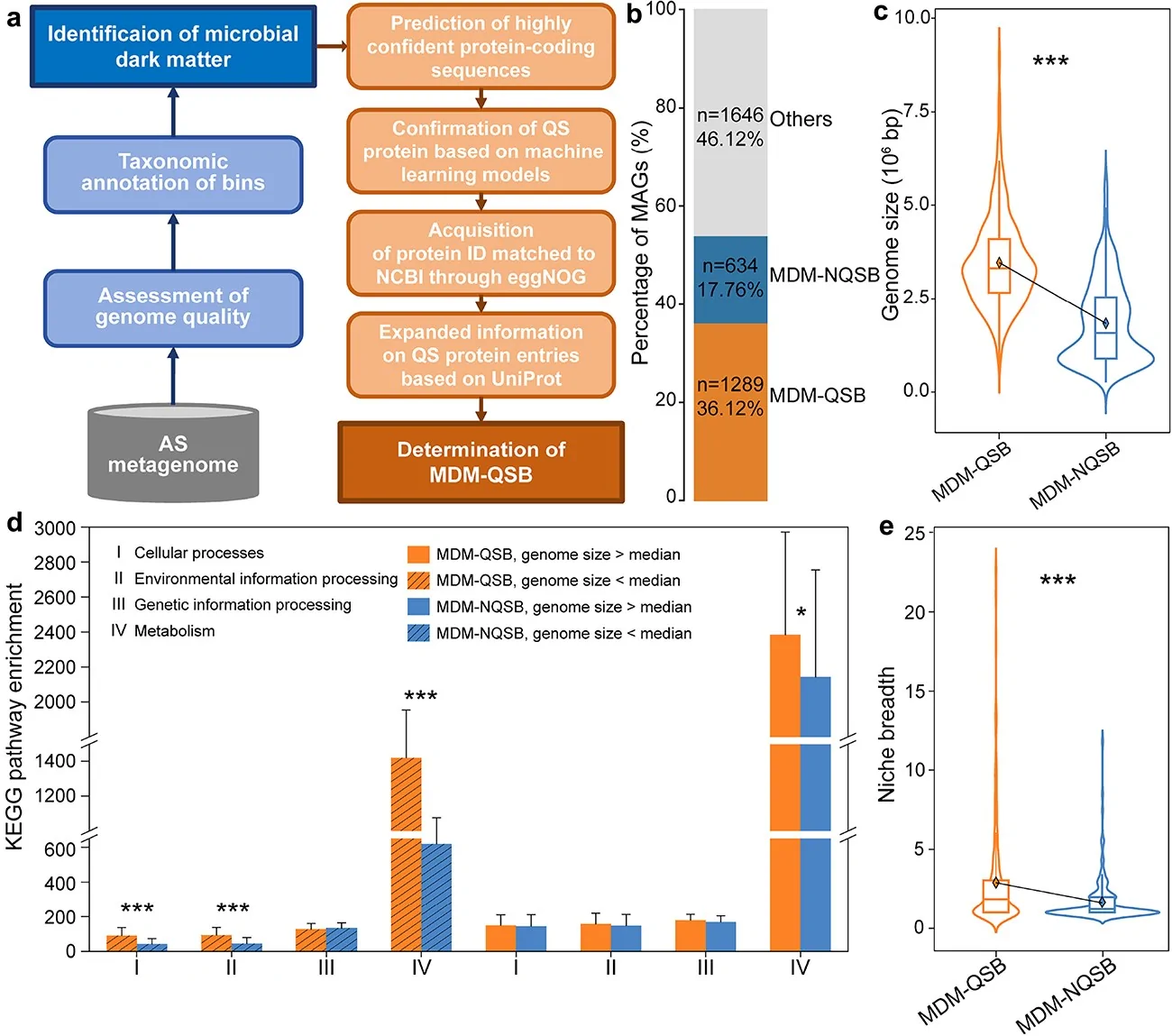
Recovery of metagenome data and genomic characteristics of MDM; (a) schematic pipeline for the identification of MDM-QSB; (b) relative representation of MDM-QSB versus non-QS MDM (MDM-NQSB) within the 3569 high- and medium-quality MAGs; (c) genome-size distribution of MDM-QSB compared with MDM-NQSB; (d) functional comparison between MDM-QSB and MDM-NQSB; cellular processes include functions such as cell growth and death, cell motility, transport, and catabolism; environmental information processing covers signal transduction, signaling molecules and interaction, and membrane transport; genetic information processing refers to DNA replication and repair, transcription, and translation; metabolism here encompasses energy metabolism and the metabolism of diverse compounds, such as amino-acid, lipid, carbohydrate, and other secondary metabolite; (e) niche-breadth estimates for MDM-QSB and MDM-NQSB.

Comparative genomic analysis demonstrated that MDM-QSB have significantly larger genome sizes (3.47 ± 1.22 Mb) than MDM-non-QSB (MDM-NQSB; 1.84 ± 1.10 Mb; *P* < .001; Fig. 1c). They also encode ~2.4 times more KEGG orthologues (5295 ± 1810 versus 2160 ± 1606; *P* < .001), reflecting greater functional potential. Genome size correlates strongly with functional diversity (*R*^2^ = 0.69, *P* < .001; Supplementary Fig. S1), suggesting that MDM-QSB possess an expanded metabolic and regulatory repertoire. In the subset of MDM-QSB with genome sizes below the median, genes associated with cellular processes such as cell growth, motility, and catabolism are significantly enriched relative to MDM-NQSB, as are genes involved in environmental information processing, which encompasses signal transduction, signaling molecule interactions, and membrane transport (Fig. 1d). This enrichment is not observed in larger MDM-QSB genomes. Across all genome size classes, however, MDM-QSB consistently exhibit higher gene counts related to energy metabolism and substance transformation. This pattern indicates a differential allocation of genomic resources that larger genomes primarily accumulate core metabolic functions, whereas smaller MDM-QSB genomes are enriched in regulatory genes involved in environmental sensing. Furthermore, MDM-QSB display a significantly broader ecological niche breadth (2.90 ± 3.33) than MDM-NQSB (1.67 ± 1.37; *P* < .01; Fig. 1e and Supplementary Fig. S2), indicating that their increased coding capacity translates into enhanced habitat versatility rather than mere genomic redundancy.

### Phylogenetic breadth and ecological prevalence of MDM-QSB

MDM-QSB comprised 1289 MAGs that resolved into 404 species, more than twice the number (195) recovered among MDM-NQSB. Consequently, MDM-QSB exhibited significantly higher species richness, Shannon diversity, and Faith’s phylogenetic diversity (all *P* < .001; Fig. 2a–c), reflecting a broad expansion of QS-proficient lineages within activated sludge. Maximum-likelihood placement of 1923 MDM MAGs revealed that MDM-QSB are distributed across 28 phyla, with *Pseudomonadota*, *Bacteroidota*, *Chloroflexota*, and *Actinomycetota* contributing the majority of sequences (Fig. 2d). The greater species count and wider phylogenetic distribution indicate that QS capability has arisen repeatedly in multiple activated sludge lineages rather than being inherited from a few ancestral generalists. QS functionality was ubiquitous in *Chloroflexota* (100% of MDM members), *Nitrospirota* (>90%), and *Cyanobacteriota* (>90%; Fig. 2e), implying that chemical communication is not an accessory trait but a core requirement for their persistence in this engineered environment. At the class level, MDM-QSB were present in 57 lineages and represented ˃50% of species in 50 of them. Among these, *Bacteroidia* and *Gammaproteobacteria* were particularly dominant. Taxonomic dissection further identified 5 novel orders, 16 families, and 127 genera lacking prior QS annotations, substantially expanding the known diversity of QS-capable microorganisms. Additionally, read-recruitment profiling showed that 90.6%–99.8% of MDM-QSB species were rare (<0.1% relative abundance per sample; Fig. 2f). Collectively, however, these rare taxa accounted for over 40% of total MDM read abundance across samples, illustrating a rare yet widespread lifestyle in which sparse populations maintain cosmopolitan distributions and contribute disproportionately to community signaling potential.

**Figure 2:**
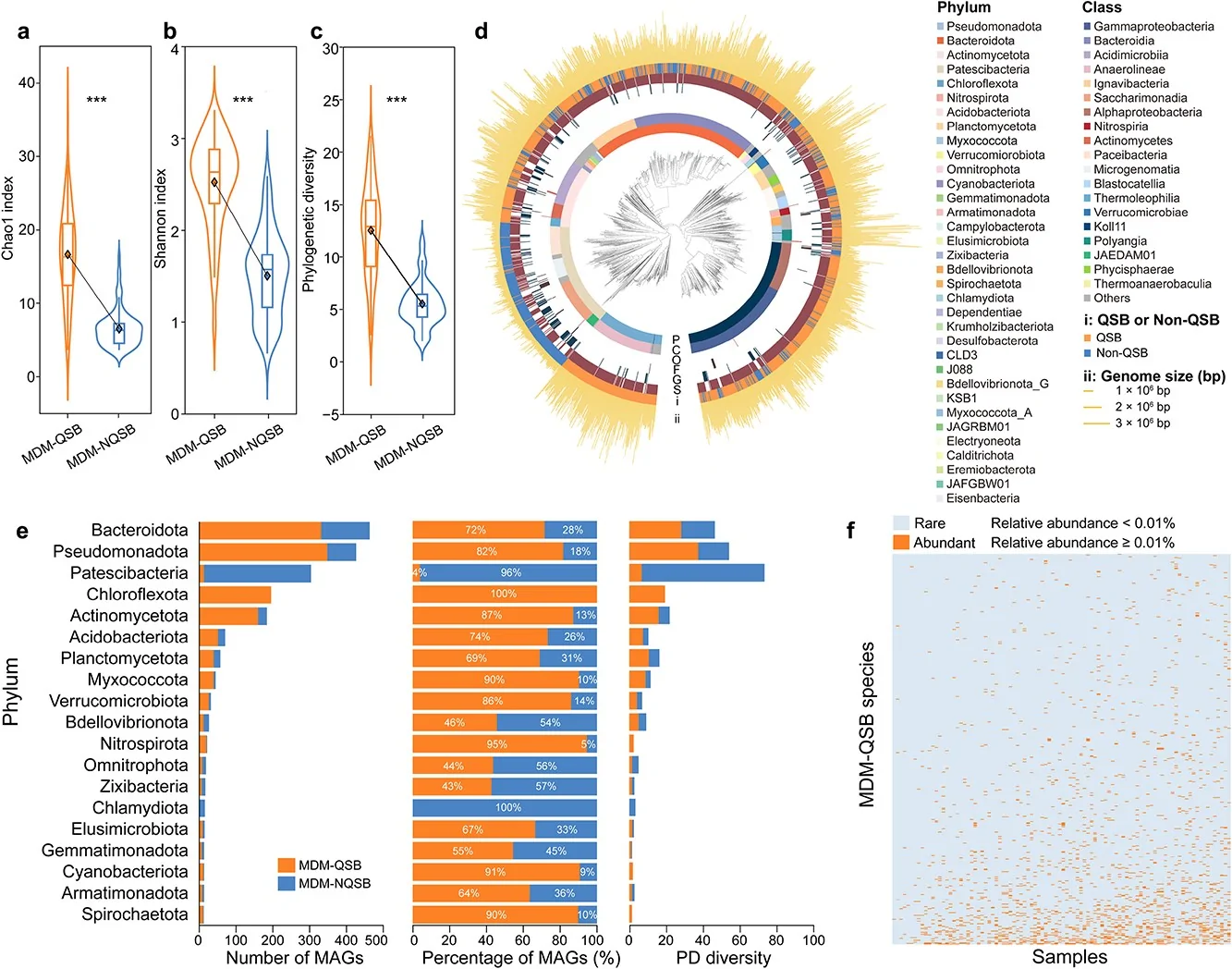
Comprehensive overview of microbial diversity and composition in MDM; comparison of (a) Chao1 index, (b) Shannon index, and (c) phylogenetic diversity between MDM-QSB and MDM-NQSB; (d) phylogenetic tree of 1923 MDM-MAGs; (e) distribution of MDM-QSB and MDM-NQSB in the top 19 phyla containing the highest number of MAGs; (f) abundance of MDM-QSB.

### Diverse quorum sensing repertoires across microbial dark matter

We mapped the QS potential of MDM-QSB across 10 classes of extracellular signaling molecules [*N*-acyl-homoserinelactones (AHLs), autoinducer-2 (AI-2), autoinducer-3 (AI-3), autoinducer peptides (AIPs), cholera autoinducer-1, dialkylresorcinols (DARs), diffusible signal factors (DSFs), 4-hydroxy-2-alkylquinolines (HAQs), indole, and photopyrone] and constructed a bipartite network linking MAGs to the signals they can produce or perceive (Fig. 3a). This analysis reveals that AHLs and AIPs are the most widely distributed QS systems, detected in over 50% of MDM-QSB species (Fig. 3b and Supplementary Fig. S3). In contrast, AI-2 and AI-3 mediate 20%–50% of interactions, while HAQs, DSFs, DARs, indole, and photopyrone are associated with fewer than 20% of taxa (Fig. 3b). Collectively, these results reveal an unexpectedly broad distribution of diverse QS mechanisms across MDM. Notably, both Gram-positive (e.g. *Actinomycetota*) and Gram-negative (e.g. *Bacteroidota*) lineages utilize all 10 signaling types.

**Figure 3:**
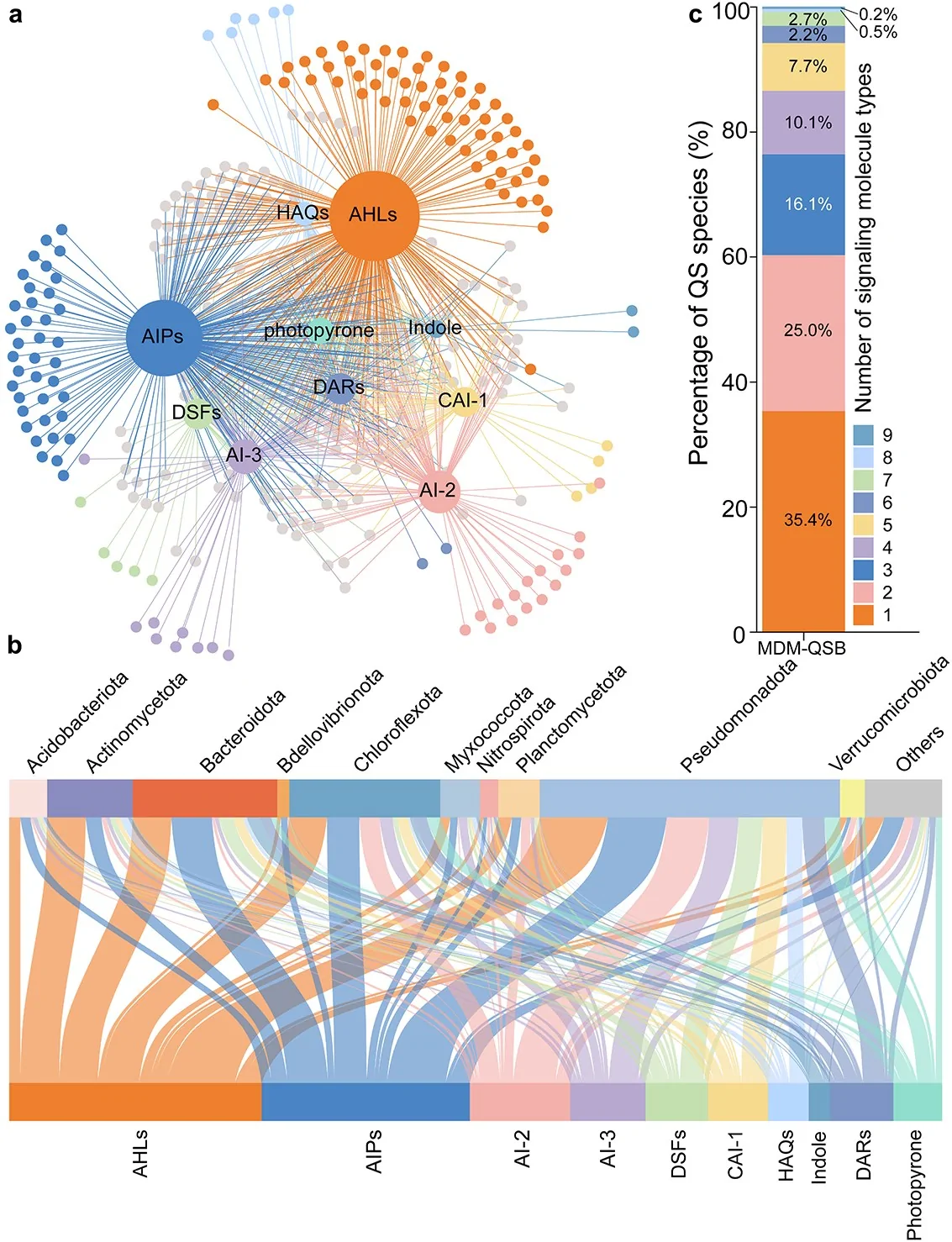
QS signaling network of MDM-QSB; (a) QS communication network composed of 10 types of signaling molecules (AHLs, AI-2, AIPs, AI-3, cholera autoinducer-1, DSF, DARs, HAQs, indole, and photopyrone); unlabeled nodes represent species, and the connection between a species and a signaling molecule indicates the capacity of the species to secrete or receive the signaling molecule; (b) utilization of various signaling molecules by different phyla; (c) the number of MDM species sharing multiple QS signaling molecules.

Furthermore, we found that ~60% of MDM-QSB encode only one or two QS systems, while 40% possess three or more (Fig. 3c). The co-occurrence of multiple signaling pathways suggests a robust communication architecture in which the loss of a single synthase or receptor can be compensated by alternative routes. Across all phyla, all 10 signal types were represented among the top 10 most abundant groups (Fig. 3b), indicating widespread distribution of diverse QS capabilities.

### Network centrality and signaling backbone roles of MDM-QSB

Co-occurrence network analyses revealed 1050 significant pairwise interactions among 397 species (*P* < .05; Fig. 4a). Nodes were ranked by within-module and among-module connectivity to identify putative keystone taxa. MDM-QSB were found to dominate this keystone set (Fig. 4a). A deep-learning classifier further confirmed their central role, placing them in the top 4% of all species in terms of structural keystoneness (Fig. 4b), a metric reflecting their influence on global network topology. Notably, even non-keystone MDM-QSB exhibited higher mean node degree (5.46) than MDM-NQSB (4.92), indicating that QS capability confers enhanced connectivity across the community, regardless of ecological prominence.

**Figure 4:**
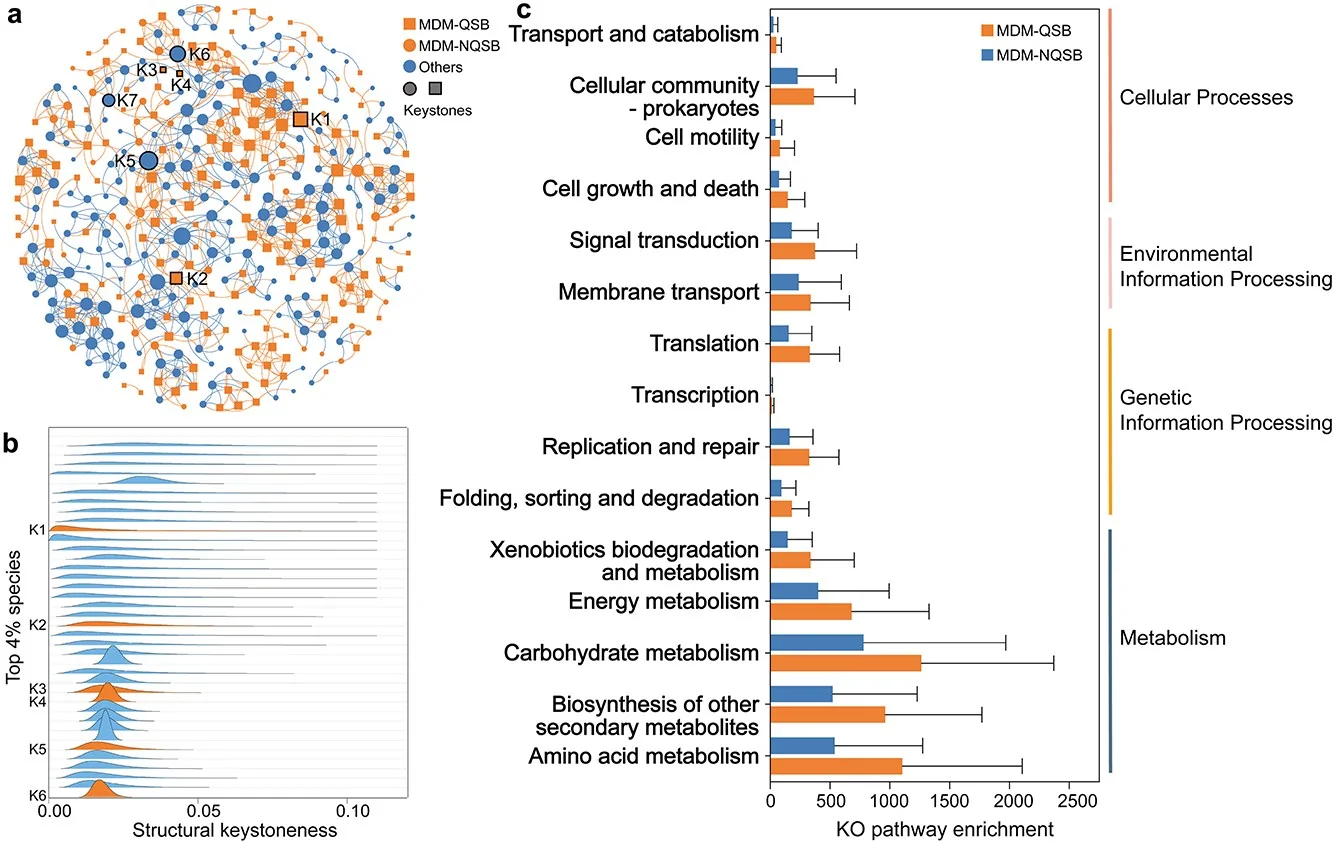
Ecological roles of MDM-QSB in species co-occurrence network; (a) co-occurrence network comprised of 397 species and 1050 connections between them; the size of each node is proportional to its node degree; nodes of the same color are affiliated with the same module; the eight main modules containing the highest number of species were colored; (b) structural keystoneness of the top 4% species identified by deep-learning model; (c) functional comparison of the intersection of top-50% co-occurrence network degree species and top-50% deep-learning-importance species.

To understand how MDM-QSB exert this dominance, we intersected the top 50% of species by node degree with those identified as high-importance by the deep-learning model, yielding a representative subset for functional profiling (Supplementary Fig. S5). These high-impact MDM-QSB showed significantly greater enrichment in environmental information processing functions compared to MDM-NQSB (Fig. 4c), including signal transduction, membrane transport, and cellular communication, functions essential for sensing and responding to extracellular cues. They also exhibited enhanced metabolic potential, particularly in energy metabolism, amino acid metabolism, and secondary metabolite biosynthesis. Additionally, these species carried elevated capacities for genetic information processing (DNA replication, transcription, and translation), suggesting a coordinated investment in both regulatory and metabolic machinery. This combination of broad metabolic versatility and advanced signaling capabilities is consistent with a role for MDM-QSB as interaction hubs that mediate regulatory signals across the community.

We further examined the organization of MDM-QSB within co-occurrence modules (Supplementary Fig. S6). The network was partitioned into eight major modules (each containing >5% of MAGs) and 22 peripheral clusters, with three modules, M4, M5, and M7, emerging as central organizers. These core modules displayed exceptional topological metrics: M5 had the highest node degree (8.4) and betweenness centrality (2180.2), M7 exhibited strong connectivity (degree = 8.18), and M4 served as a key bridge (betweenness = 1238.9). Functional analysis revealed that these core modules are enriched in genes involved in signal transduction and cellular community–prokaryotes. Specifically, the signal transduction category encompasses 32 distinct signaling pathways, while the cellular community module contains core QS components. These two functional groups are pivotal in determining the extent of QS activity within bacterial communities. Importantly, M4, M5, and M7 showed significantly higher abundance of these genes compared to other modules. Moreover, they exhibited enhanced membrane transport capacity, enabling efficient import and export of signaling molecules. As MDM-QSB constitute the majority of nodes in these three modules (Supplementary Fig. S6), their dual role in metabolic flexibility and signal mediation enables them to modulate community-wide responses to environmental perturbations.

### MDM-QSB as multifunctional hubs in microbial community

Functional analysis revealed that MDM-QSB exhibit significantly higher gene abundances across all major biogeochemical cycles such as carbon oxidation, methanogenesis, organic nitrogen metabolism, denitrification, sulfate reduction, and phosphorus cycling, compared to MDM-NQSB (Fig. 5a). MDM-QSB also show elevated abundances of genes related to nutrient mineralization and polyphosphate synthesis, suggesting enhanced metabolic versatility. These functional differences are reflected in community-level performance. Microbial communities enriched with MDM-QSB display significantly higher MIC values for COD, TN, and NH_3_-N removal (Fig. 5b). The close correlation between MDM-QSB and treatment performance suggests that the QS mechanism encourages a synergistic improvement in effluent quality at the community level.

**Figure 5:**
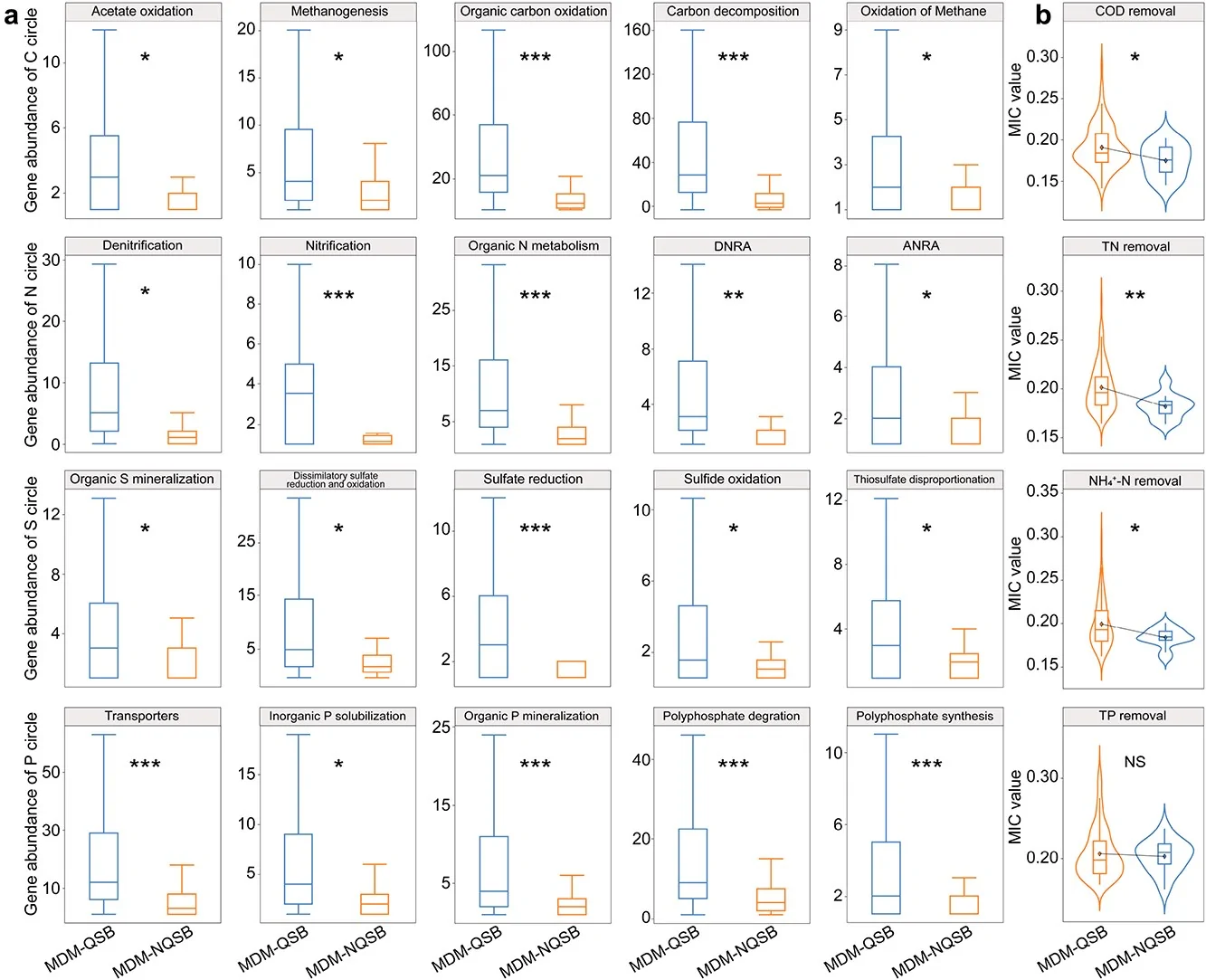
Functional analysis of MDM-QSB and MDM-NQSB; (a) functional comparison of element cycling between MDM-QSB and MDM-NQSB; DNRA represents dissimilatory nitrate reduction to ammonium; ANRA indicates assimilatory nitrate reduction to ammonium; (b) MIC between microbial community and the removal rate of COD, TN, NH_3_-N, and TP.

To elucidate the mechanisms underlying this functional dominance, we constructed a genome-scale metabolic complementarity network based on predicted metabolic exchanges between MDM-QSB and key functional populations (Fig. 6a). The network reveals extensive cross-feeding interactions between MDM-QSB and core nutrient-cycling taxa, including nitrifiers, denitrifiers, and PAOs. MDM-QSB serves as a central donor, supplying 28 distinct metabolites to these functional groups. These metabolites include three major categories: amino acids and peptides (e.g. L-serine, 4-aminobutanoate), organic compounds with specific functional roles (e.g. protoheme, choline), and inorganic ions (e.g. phosphate, calcium, sodium). We further examined the interaction between a representative MDM-QSB species (g_JAIBAC01_s_undefined), which exhibits the highest metabolite exchange flux in the network, and a nitrite-oxidizing bacterium affiliated with *Nitrospira* (g_*Nitrospira*_D_s_*Nitrospira*_D_sp002254365; Fig. 6b). This MDM-QSB species secretes QS signaling molecules, which are predicted to be detected by the nitrifier, potentially leading to transcriptional upregulation of *amoABC* and *hao*. In addition to signal-mediated regulation, the MDM-QSB exports protoheme and serine, supporting the metabolic activities of the recipient. Together, these exchanges demonstrate that MDM-QSB functions not only as a signaling mediator, but also as a metabolic donor that modulates gene expression in partner organisms.

**Figure 6:**
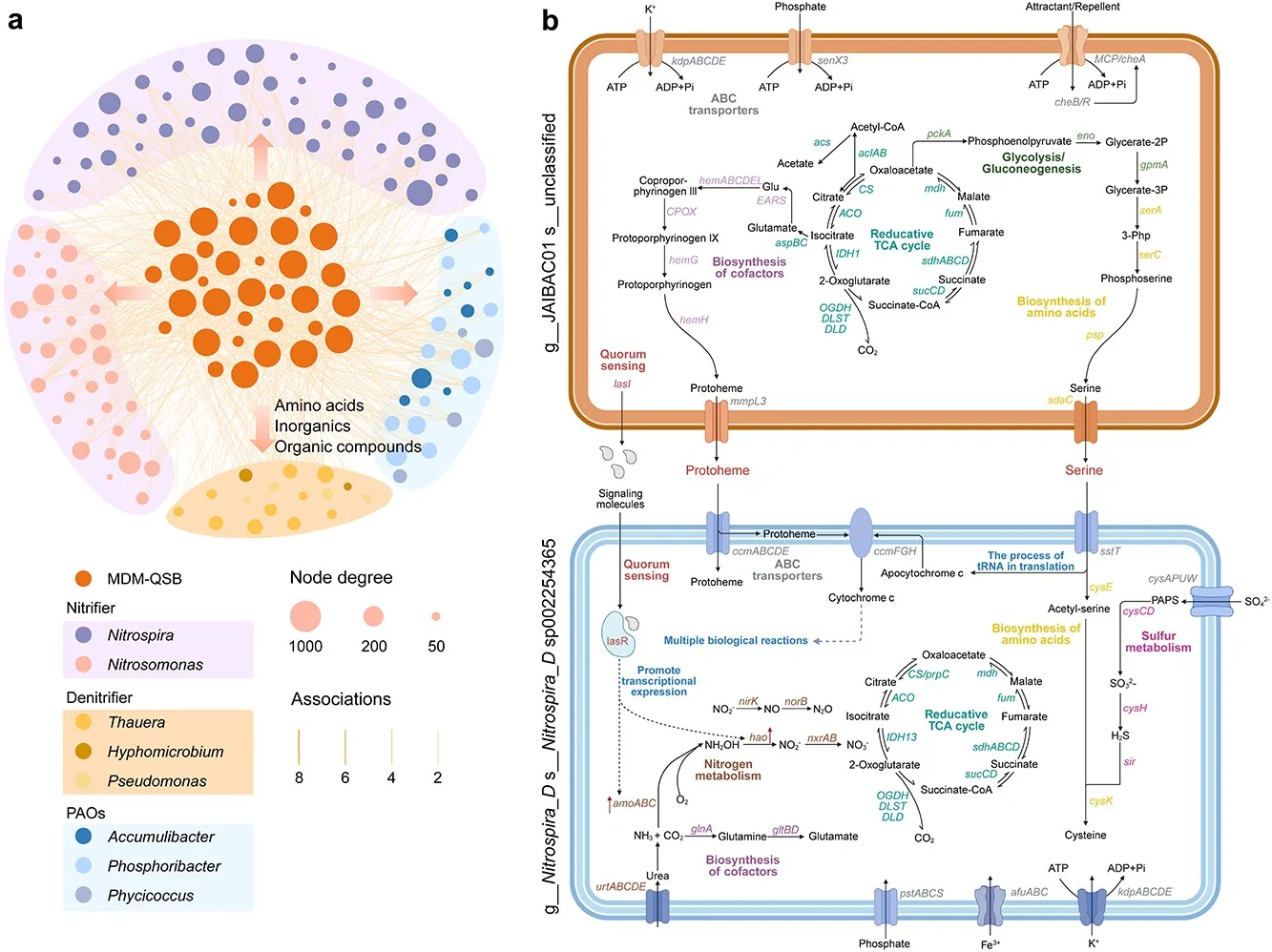
Metabolic complementarity interactions between MDM-QSB and key functional microbial groups in activated sludge; (a) metabolic complementarity network constructed from genome-scale metabolic models; nodes represent microbial species, with the central orange node denoting MDM-QSB and the surrounding nodes enclosed by shaded backgrounds representing functional nitrogen- and phosphorus-removal taxa, including nitrifiers, denitrifiers, and PAOs; node size reflects degree of connectivity, while edge thickness indicates strength of metabolic associations; (b) metabolic exchanges between a MDM-QSB strain (g_JAIBAC01_s_undefined) exhibiting the highest metabolite exchange flux in the metabolic complementarity network and a representative nitrifying species (g_*Nitrospira*_*D*_s_*Nitrospira*_*D*_sp002254365); the MDM-QSB provides the nitrifier with QS signaling molecules, protoheme, and serine.

### Discussion

The discovery of QS-capable MDM advances our understanding of community coordination in engineered microbiomes. Previous studies have established that MDMs constitute ˃50% of the total microbial abundance in activated sludge systems, yet their functional roles have remained largely enigmatic [2, 8]. Far from being passive bystanders, these organisms emerge as important contributors to both structural integrity and functional performance in activated sludge. Our analysis reveals that the majority of MDMs harbor QS capability (Fig. 1b), and receptor-only configurations predominate across diverse lineages (Fig. 1c). This overwhelming prevalence of signal-receiving capacity indicates a widespread eavesdropping strategy, wherein cells perceive exogenous autoinducers without synthesizing signals themselves [40]. Such low-cost participation enables sparse populations to synchronize behavior with the broader community while avoiding the energetic burden of signal production, thereby supporting their persistence in resource-limited and dynamically fluctuating environments. Critically, this capacity suggests that MDM-QSB acts as active participants in community decision-making through signal reception alone.

The genomic architecture of MDM-QSB further underscores their adaptive sophistication. Compared to non-QS MDMs, MDM-QSB possess significantly larger genomes and expanded functional repertoires, reflecting a dual survival strategy. Genome expansion supports metabolic versatility, while strategic enrichment of regulatory genes, particularly in smaller-genome lineages (Fig. 1d), enables high-efficiency environmental sensing under minimal replicative cost. This differential allocation of genomic resources allows even genomically constrained MDM-QSB to maintain responsiveness to chemical cues, representing an adaptive strategy under fluctuating conditions [41]. Together with their significantly broader ecological niche breadth (Fig. 1e), this genomic flexibility enables MDM-QSB to thrive across heterogeneous microenvironments, positioning them as ecological generalists. It is acknowledged that genome fragmentation could theoretically lead to false-negative identification of specific QS loci, by which the prevalence of MDM-QSB reported here may be regarded as a conservative estimate. However, given the high conservation of QS protein domains across diverse lineages [42, 43], the probability of complete omission of QS genes can be substantially reduced.

Phylogenetically, QS capability is widespread across 28 phyla (Fig. 2d and e). Remarkably, even within well-studied phyla such as *Pseudomonadota* and *Nitrospirota*, which have long been regarded as model systems for QS research [44, 45], 43% and 21% of their QS-competent members remain unclassified at the species level (Supplementary Fig. S4). This residual QS dark matter underscores the incompleteness of current taxonomic frameworks and highlights the necessity of genome-resolved metagenomics to uncover cryptic signaling functions. The pervasive presence of rare MDM-QSB establishes a persistent background of signal perception across diverse microenvironments (Fig. 2f). This pattern supports the concept of background sensing, whereby low-abundance populations detect shared signaling molecules and modulate gene expression without dominating community biomass [1, 46]. Such a configuration enables rapid, community-wide metabolic adjustments to environmental perturbations without requiring compositional shifts in dominant taxa. In addition, this hidden prevalence corrects a long-standing underestimation of the scope of QS in engineered systems. Previous 16S ribosomal RNA (rRNA) gene surveys estimated QSB prevalence at ~14% in wastewater treatment systems [47], while another study cataloged around 985 QS-positive genera based on reference databases [48]. However, these approaches suffered from limited genomic resolution and an inability to distinguish signal producers from signal responders. In contrast, our MAG-based protein annotation reveals a far more extensive QS landscape, encompassing both signal synthesis and perception across the full phylogenetic breadth of MDM. Excluding MDM-QSB from communication models likely leads to substantial underestimation of both the scale and ecological relevance of QS in activated sludge ecosystems. Therefore, by revealing a previously hidden layer of signal-responsive organisms, our findings may help explain the unexpected performance variability in conventional monitoring approaches that focus exclusively on abundant, classified lineages [49, 50].

MDM-QSB encode components for all 10 major classes of QS signaling molecules (Fig. 3a), reflecting a broad molecular repertoire. These signaling systems are distributed across phylogenetically distant lineages, including those typically classified as Gram-positive and Gram-negative, indicating that QS molecule usage is not strictly constrained by phylogeny. This broad cross-phylum utilization supports growing evidence that QS systems traditionally considered intraspecific can function in interspecies communication. Such cross-talk has been proposed in human gut microbiomes [51] and is now demonstrated in engineered ecosystems, suggesting that shared signaling languages may facilitate community-wide coordination across phylogenetic boundaries. Moreover, 40% of MDM-QSB encode three or more QS pathways (Fig. 3c), suggesting a redundant communication architecture that enhances the resilience of community-wide coordination under the chemically complex and fluctuating conditions typical of wastewater treatment systems.

The redundancy of MDM-QSB corresponds to their topological prominence in microbial co-occurrence networks. The central role of MDM-QSB in the activated sludge microbiome was identified through two complementary approaches (Fig. 4a and b): microbial co-occurrence network analysis and the deep learning-based embedding model cNODE2. The co-occurrence network provides interpretable insights into potential ecological interactions, while cNODE2 captures higher-order topological features beyond pairwise correlations [31]. By intersecting the outputs of both methods, we mitigated the inherent uncertainty associated with correlation-based inference alone [52]. MDM-QSB not only occupy keystone positions, but also constitute the majority of nodes in core modules (Supplementary Fig. S6). Elevated node stress values (M5 = 556481.7; M7 = 357334.3; M4 = 1238.9; Supplementary Fig. S6) suggest these modules act as primary information brokers, facilitating cross-module communication, and resource flow [53, 54]. Genomic analyses reveal enrichment in 32 distinct signal transduction systems, along with cellular communication components and membrane transport machinery, supporting their capacity for rapid uptake, processing, and dissemination of autoinducers (Fig. 4c).

Functionally, MDM-QSB extend beyond signaling roles to act as multifunctional metabolic hubs. They exhibit elevated gene abundances across all major biogeochemical cycles (Fig. 5a), and activated sludge communities with higher MDM-QSB abundance show significantly improved removal efficiencies for COD, TN, and NH_3_-N (Fig. 5b). Previous studies have widely reported that QSB enhance nitrogen removal by stimulating the expression of key functional genes through signaling molecules, thereby reinforcing downstream nitrogen transformation processes [55, 56]. Consistent with this established mechanism, our analysis predicts that QS signals produced by MDM-QSB are detectable by nitrifiers such as *Nitrospira*. Based on genomic context and regulatory motif conservation, these signals are associated with the upregulation of *amoABC* and *hao* [57], which encode ammonia monooxygenase and hydroxylamine oxidoreductase, respectively. These enzymes catalyze critical steps in aerobic ammonia oxidation and support subsequent nitrogen conversion [58, 59]. It should be noted, however, that this regulatory link is inferred from genomic context and the conservation of regulatory motifs rather than from direct expression evidence, and thus remains a hypothesis to be validated experimentally, e.g. through metatranscriptomics or stable-isotope probing.

Beyond this known signaling-mediated regulation and the intrinsic degradation capacity of QSB, our GSMM reveals a previously underappreciated mechanism: extensive cross-feeding interactions between MDM-QSB and key functional guilds, including nitrifiers, denitrifiers, and PAOs (Fig. 6a). For example, the MDM-QSB species g_JAIBAC01_s_undefined is predicted to synthesize protoheme and serine, both of which are essential but cannot be synthesized *de novo* by a co-occurring *Nitrospira* population (Fig. 6b). Protoheme serves as a direct precursor for cytochrome c biosynthesis in the nitrifier, which is critical for electron transfer during aerobic respiration [60]. Serine contributes to cysteine biosynthesis, supporting redox homeostasis and maintaining enzyme functionality in nitrogen-metabolizing pathways [61]. Thus, these results suggest that metabolic cross-feeding from MDM-QSB to canonical nitrifiers ensures essential physiological functions in the latter and directly enhances nitrogen metabolism. By combining metabolite provisioning with signal-mediated regulation, MDM-QSB bridge metabolic and signaling networks. By simultaneously supplying indispensable metabolites and modulating gene expression in partner taxa, MDM-QSB stabilize and enhance the activity of otherwise specialized microbes, thereby contributing to the overall efficiency of nutrient removal in activated sludge ecosystems. However, this study’s limitations should be acknowledged. Although our analyses provide evidence and mechanistic plausibility, the causal roles of MDM-QSB in community coordination and nutrient removal remain to be further experimentally validated using approaches such as metatranscriptomics, stable isotope probing, or targeted enrichment experiments. Nevertheless, the consistency across multiple independent analytical approaches lends robust support to our central findings.

In conclusion, this study suggests a previously underappreciated role: MDM-QSB are associated with extensive cross-feeding of essential metabolites with key functional taxa involved in nitrification, denitrification, and phosphorus cycling. This metabolic interdependence, combined with the ability to modulate partner gene expression through QS signals, positions these organisms as putative dual-function hubs that integrate regulatory communication with obligate nutritional support. This reframing offers a basis for improving our capacity to model, monitor, and control biological wastewater treatment processes. Moreover, despite their typically low relative abundance, MDM-QSB are consistently enriched in high-performing systems across diverse full-scale WWTPs, suggesting their potential as future targets for bioaugmentation or process optimization once causal relationships are established through controlled experiments.

## Supplementary Material

Supplementary_materials_ycag264

## Data Availability

Additional figures (Supplementary Figs S1–S6) and table (Supplementary Table S1) are available in the Supplementary Material. Sequence data associated with this project have been deposited in the NCBI Database with accession number PRJNA1271453 (https://www.ncbi.nlm.nih.gov/search/all/?term=PRJNA1271453).
