# Supplementary material for: Quorum-sensing microbial dark matter enables core functions in wastewater microbiomes
Source: ISME Commun. 2026 Sep 16;6(1):ycag264. doi: 10.1093/ismeco/ycag264 (PMC13641062; doi:10.1093/ismeco/ycag264)
Supplement: Supplementary file 1 [file supplementary_materials_ycag264.docx]

Supplementary Information for

Quorum-sensing microbial dark matter enables core functions in wastewater microbiomes

Qiuju Liu^1^, Han Su^1,2^, Sai Gong^1^, Ying Jin^1^, Yingzheng Fan^1^, Mengxiong Wu^1^, Jianhua Guo^3^, Xinkun Ren^4,^*****, Jinfeng Wang^1,^*****

^1^ State Key Laboratory of Water Pollution Control and Green Resource Recycling, School of the Environment, Nanjing University, Nanjing 210023, Jiangsu, P.R. China

^2^ Institute for the Environment and Health, Nanjing University Suzhou Campus, Suzhou 215163, Jiangsu, P.R. China

^3^ Australian Centre for Water and Environmental Biotechnology (ACWEB, formerly AWMC), The University of Queensland, St Lucia, QLD 4072, Australia

^4^ College of Engineering and Applied Sciences, Nanjing University, Nanjing 210023, Jiangsu, P.R. China

* Corresponding Author

Xinkun Ren, Jinfeng Wang

E-mail: [xinkunren@nju.edu.cn](mailto:xinkunren@nju.edu.cn); [jfwang@nju.edu.cn](mailto:jfwang@nju.edu.cn)

Contents

[Fig. S1. The correlation between genome size and the number of functional genes.	3](#_Toc235145374)

[Fig. S2. The number of species in different ecological niches.	4](#_Toc235145375)

[Fig. S3. Comparison of the number of QS-related species and proteins in MDM and Non-MDM. (A) Number of QS species in MDM and Non-MDM. (B) Number of QS protein entries in MDM and Non-MDM.	5](#_Toc235145376)

[Fig. S4. Distribution of MDM and non-MDM in the top 20 phyla containing the highest number of MAGs.	6](#_Toc235145377)

[Fig. S5. Keystone species identified by both the co-occurrence network (the top 50% of species ranked by node degree) and the deep-learning model (the top 50% of species ranked by structural keystoneness).	7](#_Toc235145378)

[Fig. S6. Modular structure of the co-occurrence network. (A) Eight major modules (each containing >5% of nodes) and 22 peripheral clusters in the co-occurrence network. (B) Functional comparison of eight major modules.	8](#_Toc235145379)

[Table S1. Geographical distribution of activated sludge samples. A^2^O, AO, IFAS, CASS, and OD correspond to anaerobic-anoxic-oxic, anoxic-oxic, integrated fixed-film activated sludge, cyclic activated sludge system, and oxidation ditch, respectively.	9](#_Toc235145380)

**Fig. S1.** The correlation between genome size and the number of functional genes.

**Fig. S2.** The number of species in different ecological niches.

**Fig. S3.** Comparison of the number of QS-related species and proteins in MDM and Non-MDM. (A) Number of QS species in MDM and Non-MDM. (B) Number of QS protein entries in MDM and Non-MDM.

**Fig. S4.** Distribution of MDM and non-MDM in the top 20 phyla containing the highest number of MAGs.

**Fig. S5.** Keystone species identified by both the co-occurrence network (the top 50% of species ranked by node degree) and the deep-learning model (the top 50% of species ranked by structural keystoneness).

**Fig. S6.** Modular structure of the co-occurrence network. (A) Eight major modules (each containing >5% of nodes) and 22 peripheral clusters in the co-occurrence network. (B) Functional comparison of eight major modules.

**Table S1.** Geographical distribution of activated sludge samples. A^2^O, AO, IFAS, CASS, and OD correspond to anaerobic-anoxic-oxic, anoxic-oxic, integrated fixed-film activated sludge, cyclic activated sludge system, and oxidation ditch, respectively.

| Sample | Province | Process | Sample | Province | Process | Sample | Province | Process |
| --- | --- | --- | --- | --- | --- | --- | --- | --- |
| s1 | Jiangsu | A^2^O | s37 | Jiangxi | OD | s70 | Hunan | A^2^O |
| s2 | Guangdong | A^2^O | s38 | Jiangxi | OD | s71 | Hunan | A^2^O |
| s3 | Guangdong | A^2^O | s39 | Jiangxi | OD | s72 | Hunan | A^2^O |
| s4 | Guangdong | A^2^O | s40 | Jiangxi | IFAS | s73 | Hunan | A^2^O |
| s5 | Guangdong | A^2^O | s41 | Jiangxi | IFAS | s74 | Hunan | A^2^O |
| s6 | Guangdong | A^2^O | s42 | Fujian | A^2^O | s75 | Guizhou | A^2^O |
| s7 | Guangdong | A^2^O | s43 | Fujian | OD | s76 | Beijing | A^2^O |
| s8 | Guangdong | A^2^O | s44 | Fujian | OD | s77 | Ningxia | OD |
| s9 | Guangdong | A^2^O | s45 | Fujian | OD | s78 | Ningxia | OD |
| s10 | Guangdong | IFAS | s46 | Fujian | AO | s79 | Ningxia | IFAS |
| s11 | Gansu | A^2^O | s47 | Fujian | AO | s80 | Ningxia | IFAS |
| s12 | Gansu | A^2^O | s48 | Shandong | A^2^O | s82 | Qinghai | IFAS |
| s13 | Gansu | IFAS | s49 | Jilin | A^2^O | s83 | Qinghai | IFAS |
| s14 | Sichuan | IFAS | s50 | Jilin | A^2^O | s84 | Hubei | AO |
| s15 | Sichuan | A^2^O | s51 | Jilin | A^2^O | s85 | Liaoning | CASS |
| s16 | Sichuan | OD | s52 | Jilin | A^2^O | s86 | Liaoning | CASS |
| s17 | Sichuan | A^2^O | s53 | Henan | AO | s87 | Liaoning | A^2^O |
| s18 | Tianjin | AO | s54 | Henan | A^2^O | s90 | Liaoning | CASS |
| s19 | Tianjin | AO | s55 | Henan | IFAS | s91 | Liaoning | CASS |
| s20 | Tianjin | AO | s56 | Henan | A^2^O | s92 | Liaoning | CASS |
| s21 | Tianjin | IFAS | s57 | Henan | A^2^O | s93 | Shanghai | IFAS |
| s22 | Hebei | IFAS | s58 | Henan | A^2^O | s94 | Yunnan | CASS |
| s23 | Hebei | A^2^O | s59 | Henan | A^2^O | s95 | Hainan | A^2^O |
| s24 | Hebei | OD | s60 | Anhui | OD | s96 | Hainan | A^2^O |
| s25 | Hebei | AO | s61 | Zhejiang | AO | s97 | Hainan | A^2^O |
| s27 | Heilongjiang | AO | s62 | Zhejiang | AO | s98 | Hainan | A^2^O |
| s30 | Xizang | A^2^O | s63 | Shaanxi | IFAS | s99 | Hainan | CASS |
| s31 | Xizang | A^2^O | s64 | Shaanxi | A^2^O | s100 | Hainan | A^2^O |
| s32 | Xizang | A^2^O | s65 | Shaanxi | A^2^O | s12_6_1 | Heilongjiang | AO |
| s33 | Xizang | A^2^O | s67 | Shaanxi | A^2^O | s12_8_1 | Heilongjiang | A^2^O |
| s35 | Jiangxi | AO | s68 | Hunan | A^2^O | s146_1 | Shaanxi | A^2^O |
| s36 | Jiangxi | OD | s69 | Hunan | A^2^O | s258_1 | Liaoning | CASS |
